# Dealing with the complex dynamics of teaching hospitals

**DOI:** 10.1186/s12909-016-0623-3

**Published:** 2016-04-05

**Authors:** Tiuri R. van Rossum, Fedde Scheele, Albert J. J. A. Scherpbier, Henk E. Sluiter, Ide C. Heyligers

**Affiliations:** Maastricht University – School of Health Professions Education, Universiteitssingel 60, 6229 ER Maastricht, The Netherlands; OLVG Teaching Hospital, VU Medical Center – Athena Research Institute, Jan Tooropstraat 164, 1061 AE Amsterdam, The Netherlands; Maastricht University Medical Centre - Faculty of Health Medicine and Life Sciences, Universiteitssingel 60, 6229 ER Maastricht, The Netherlands; Deventer Hospital –Internal medicine and nephrology, Nico Bolkesteinlaan 75, 7415 SE Deventer, The Netherlands; Zuyderland MC Teaching Hospital, Maastricht University - School of Health Professions Education, Universiteitssingel 60, 6229 ER Maastricht, The Netherlands

**Keywords:** Postgraduate medical education, Competency based medical education, Flexible medical education, Change management, Implementation, Complexity theory, Diffusion of innovations theory, Teaching hospital, Organizational science

## Abstract

Innovation and change in postgraduate medical education programs affects teaching hospital organizations, since medical education and clinical service are interrelated.

Recent trends towards flexible, time-independent and individualized educational programs put pressure on this relationship. This pressure may lead to organizational uncertainty, unbalance and friction making it an important issue to analyze.

The last decade was marked by a transition towards outcome-based postgraduate medical education. During this transition competency-based programs made their appearance. Although competency-based medical education has the potential to make medical education more efficient, the effects are still under debate. And while this debate continues, the field of medical education is already introducing next level innovations: flexible and individualized training programs. Major organizational change, like the transition to flexible education programs, can easily lead to friction and conflict in teaching hospital organizations.

This article analyses the organizational impact of postgraduate medical education innovations, with a particular focus on flexible training and competency based medical education. The characteristics of teaching hospital organizations are compared with elements of innovation and complexity theory.

With this comparison the article argues that teaching hospital organizations have complex characteristics and behave in a non-linear way. This perspective forms the basis for further discussion and analysis of this unexplored aspect of flexible and competency based education.

## Background

### Innovating postgraduate medical education

In the endeavor to provide good Post Graduate Medical Education (PGME), the field of medical education is in continuous development. Implementing innovations and change in PGME curricula is a common and continuous process. An innovation that is introduced on a worldwide scale is competency-based medical education (CBME). CBME makes it possible to make medical training more outcome-based [1]. It may guarantee more accountable outcomes and the achievement of pre-set goals and standards, which makes it an attractive educational innovation [2–4].

While the search for better education has to be encouraged, the implementation of innovations and change in PGME programs affects teaching hospital organizations, since medical education and clinical service are interrelated. Traditionally, PGME takes place in a workplace setting [5], where residents also play an important role in clinical service. Implementation of innovations and change in PGME programs therefore does not only influence education, but also has an impact on teaching hospital organizations as a whole. Organizational change in this context may be expected to lead to unexpected challenges, friction, uncertainty, and unsettled feelings in teaching hospitals organizations [3].

### CBME and Individual training programs

One of the recent trends in medical education that exerts pressure on the relationship between education and teaching hospital organizations is the emergence of time-independent educational programs. This tendency towards individualized and flexible training programs is partly due to resident work hour restrictions and maternity leave, but it is also a consequence of the transition to CBME and flexible education programs.

CBME arises from a tendency to provide more outcome-based medical education [1] and stresses the need to train modern doctors, who are able to meet societal needs [6–8]. Competency based frameworks are forming the basis of medical education in the Western world [7, 9, 10]. The basis of CBME can be constructed around four central themes: based on outcomes, a focus on abilities rather than knowledge, an emphasis on the attainted learning rather than time spend on learning and learner centeredness [7].

### Individual training programs

When moving to outcome-based education, the required time to reach these desired outcomes comes under debate, since residents reach competencies at different paces. To do justice to individual learning curves and to acknowledge residents’ individual qualities, curricula can become flexible and individualized by uncoupling competencies and education time [6].

From an educational point of view, this development is reasonable, but the emergence of flexible training programs are a reality that inevitably leads to organizational challenges that professionals have to deal with. It may easily lead to complex problems in terms of scheduling residents, making assessments at different times, financial consequences, and assuring educational standards. These organizational effects should not be underestimated when introducing and implementing educational innovations.

How these effects will exactly manifest in teaching hospitals is yet to be discovered. To understand organizational processes caused by the flexibilisation of PGME training programs that are currently introduced in teaching hospitals worldwide requires a suitable framework that captures the complex dynamics of a teaching hospital.

In this perspective, we aim to enrich the discussion about the implementation of CBME and the emergence of flexible curricula with insights from complexity theory. In the literature about innovation and change in medical education, many studies have taken the perspective of the diffusion of innovations theory [11, 12] Several studies used this theory to analyze the implementation of educational innovations, such as new assessment tools [13] and the introduction of Entrustable Professional Activities (EPAs) [14]. Although the diffusion of innovation theory is an important theory, we argue that to fully understand the effects of the proposed changes in PGME and to grasp the full organizational complexity of teaching hospitals we have to draw additional insights from complexity theory.

## Discussion of conceptual theories

### Diffusion of innovation characteristics

Rogers’ diffusion of innovations theory provides a widely used perspective on the diffusion and adoption of innovations in social groups and organizations [11]. The theory states that the adoption of innovations in organizations is affected by various factors [15]. Rogers’ theory originates from rural sociology, and his description of the concept is based on his research on the adoption process of innovations in farming [15]. The diffusion process was described as the spread of new ideas and innovations that mostly happens due to imitation [15]. He described several factors and interventions that influence this process. The four most important influencing factors are the relative advantage of the innovation, system readiness, user readiness and resource readiness.

### The relative advantage of an innovation

The adoption of an innovation is affected by the characteristics of the innovation itself [15]. Evidence suggests that simple innovations are more easily implemented than more complex innovations [16]. Furthermore, innovations with a relative advantage are more easily implemented than innovations that lack such an advantage [11, 17]. This is reinforced if the effects of an innovation are easily observable [18]. However, there is also empirical evidence that a relative advantage does not guarantee direct adoption [16].

Additionally, adoption is influenced by the fit of the norms, values and needs in the organization [16] as well as by the availability of knowledge that is required for the innovation [19].

### System readiness for innovation

The adoption of innovations is also influenced by organizational structures, culture and context [15]. There are several distinguishable organizational features that help adopting new innovations, such as the presence of a long-term strategy, strong leadership and good managerial relationships [20]. Taken together, these features are important determinants of what Rogers called system readiness for innovation. Conversely, organizational barriers that hinder implementation are characterized by the opposite features. Hence the organization needs to be willing and ready to adapt to an innovation [15].

### User readiness

User readiness is defined as the extent to which the users are ready and able to apply the innovation in their day-to-day activities. Staff members are not passive recipients of innovations; they experiment with them, work with them, challenge them, evaluate them, and find meaning in them [15]. Rogers speaks of the desirable, undesirable, direct, indirect, anticipated, and unanticipated effects that innovations have on the users in the organizational system [11]. These direct and indirect effects have to be experienced in order to experience if innovations have a positive effect on day-to-day activities. When innovations have these effects they are more easily adopted.

### Resource-readiness

Finally the innovation has to fit into the organizational resource system, which means that there has to be dedicated resources that can be addressed for the innovation and the innovation process [21–23]. Resource readiness is the state in which there are sufficient resources present that make the implementation and effectuation of an innovation possible. This is mainly reflected in the financial capability of an organization, but resources can also include the availability of time, knowledge and expertise. An innovation is not likely to be implemented in an organization that is lacking the required resources for the innovation or the implementation process, in other words if it is lacking resource-readiness.

### Reflection on innovation theory characteristics

In innovation theory, the innovation and adoption processes are the key elements, while the organization in which the innovation is implemented is approached as a single delimited entity. This is reflected in the factors that are assumed to play a role in the adoption process. While we consider all these factors to be very important, if not essential, for the dissemination of innovations, we believe that they cannot fully explain the effects of an innovation in the specific case of medical education, because the organization of a teaching hospital adds complexity to the theory described by Rogers [11].

So far, studies on innovations in medical education that used the dissemination of innovation theory as a theoretical framework approached each teaching hospital organization as a single system that interacts with the innovation. In reality, this theoretical approach probably does not do justice to the many interactions that exist within the different parts of an organization, and particularly in a teaching hospital. Teaching hospitals are divided into many different organizational parts, such as different administrative levels, wards and out-patient-clinics, which all function individually as well as interdependently over time. Another factor adding complexity to the interactions between these parts is their cultural diversity.

We also believe that in the case of innovation in medical education, a crucial additional complicating factor in the outcome of the adoption process is the interconnectedness between PGME and teaching hospital organizations. In teaching hospitals, educational challenges have to be integrated in the clinical processes [24]. Due to this interconnectedness, we believe that teaching hospitals are organizations that are possibly more complex than the farming organizations on which the diffusion of innovation theory was based. Therefore we suggest broadening our perspective with insights from complexity theory.

### Complexity theory characteristics and teaching hospital organization

Complexity theory suggests that organizations consist of multiple interacting parts that are called ‘systems’. This means that an organization cannot be seen as a clearly defined and delimited entity; rather, it has to be approached as an organism with different interacting parts. To understand these sub-systems, they have to be analyzed in the context of, and in relation to, other systems rather than in isolation. Systems need to be observed and described to get a better grip on what happens when variables in these systems are changed [25] as well as to discover overall patterns and mechanisms within the system [26].

In the case of innovation in medical education, this means that innovation and change in medical education has effects on other parts of the organization. Therefore, to fully understand organizational change in teaching hospital organizations, we have to analyze the organization as a whole, including the clinical service, instead of limiting our perspective to the educational part of the organization. The following sections will describe the main characteristics of complex organizations: non-linearity, co-evolution, fuzzy boundaries and actors acting in different systems.

### Non-linearity and co-evolution

Complex systems often behave in a non-linear way; this means that organizational change can have disproportional effects in relation to the delivered input. These effects can be exponential and could take place in other systems of the organization than the system in which the intended change was desired [27]. This organizational behavior makes complex systems sensitive to small changes [27], and a given change may, at the same time, have predictable and unpredictable effects [24].

In teaching hospitals there are indications that the different systems do not only interact, but are also integrated in each other. For instance, PGME and clinical service are thus far so intricately embedded that it is impossible to provide PGME without delivering clinical service. Embedded systems – like PGME in teaching hospitals – often show signs of co-evolution [28]. If one of the embedded systems changes, this simultaneously affects the other systems and vice versa. This process often leads to frictions and tensions between these systems. Co-evolution makes it therefore difficult to analyze each system separately without reference to the other systems [25].

One of the tensions that the introduction of flexible PGME programs causes by co-evolution is that clinical service can no longer be modeled around standardized training programs. A transition to flexible CBME programs will make the time that residents are available more flexible, leading to an uncertain workflow and making it difficult to schedule clinical service.

This might have a negative effect on clinical staff members’ drive to invest in residency training. In a clinical setting that is already characterized as overstrained and overworked, such an additional challenge may result in less motivated clinical teachers and might trigger feelings of exhaustion, or even burnout. While this may be an extreme example, it underlines the need to consider the interaction and between, and embedding of PGME and other systems in teaching hospital organizations.

### Fuzzy boundaries, and actors acting in different systems

Traditional systems have clearly defined organizational boundaries. These fixed boundaries make it possible to view organizations as delimited systems that interact with an external environment [25]. By contrast, complex systems have less clearly defined and more ‘fuzzy’ boundaries. In complex systems, it is difficult to separate different interacting systems [25]. This is reinforced y the professional actors working in these organizations: in complex organizations, the same professionals will operate – sometimes simultaneously - in different systems. This is certainly the case in the clinical process of teaching hospitals where doctors have several roles at the same time. For instance, during a surgical procedure the surgeon delivers patient care and acts as an educator, while he or she can be an entrepreneur and a manager at the same time. Actors that simultaneously act in multiple systems reinforce the interaction and embedding of these systems. In most teaching hospitals, professionals simultaneously act in the different systems, and these systems are embedded in each other. For instance, for some clinical tasks residents receive direct feedback from supervisors, but residents also perform clinical tasks with lesser amounts of supervision. The same goes for the supervisors, who can demonstrate their skills to residents while delivering clinical service, but who also must deliver the same clinical service without an educational component, in order to meet the expectations of other interested stakeholders, such as patients, the administration of the institution and health care authorities. The co-existence of multiple, embedded, systems complicates the implementation of innovation and of changes in educational systems and can lead to unexpected effects in organizations [25].

## Conclusions

Postgraduate Medical education is undergoing rapid changes, making innovation and change in PGME curricula a common and continuous process. The urge for more accountable PGME has led to the introduction of competency-based frameworks in medical education, leading to the current transition to flexible and individualized curricula. Besides the educational effects, these innovations and changes also have an impact on the organization of teaching hospitals.

Based on our conceptual reflection we can draw several conclusions. We started our discussion with a description of the diffusion of innovations theory. Although this theory has a strong basis and is often used when studying innovation and assimilation in organizations, we argue that the specific organization of a teaching hospital has a number of characteristics that are best described in combination with complexity theory. In the case of PGME and the transition to flexible and individualized training programs, we identified several indicators which show that teaching hospital organizations have complex characteristics.

Since PGME is fully embedded in the clinical service of a teaching hospital, changing PGME has direct consequences for other aspects of delivering clinical service in a teaching hospital. Agents in medical education, i.e. the clinical staff and residents, are acting in different parts of the organization. Changes in PGME programs affect the involved agents and can have effects on other parts of the organization. This can lead to unexpected effects in response to change. These non-linear effects in complex organizations can complicate change processes. We recommend that these characteristics, based on complexity theory, have to be taken into account when changing medical education

### Practical implications and Future research

In terms of practical implications, the insights discussed in this paper can be used for implementation and change processes in medical education. First of all, when implementing innovations or making organizational changes, formal change management strategies seem to be a good starting point.

Secondly, because of the complex setting of teaching hospitals, organizational change can only be initiated by involving stakeholders that are involved in medical education as well as stakeholders from other organizational systems. This means that in practice there has to be a great awareness of the organizational systems and of the stakeholders involved with postgraduate medical education. A way of dealing with this complexity is to make a thorough stakeholder analysis.

Lastly we recommend that for future research this complexity should be taken into account when exploring the mechanisms that influence the organization of teaching hospitals when fundamental changes are made in PGME.

### Ethics approval and consent to participate

‘Not applicable’.

### Consent for publication

‘Not applicable’.

### Availability of data and materials

Not applicable, no data besides the literature in the reference list was used for this study.

## Abbreviations

CBME: competency based medical education
EPA: entrustable professional activities
PGME: postgraduate medical education
